# PMMA bone cement containing long releasing silica-based chlorhexidine nanocarriers

**DOI:** 10.1371/journal.pone.0257947

**Published:** 2021-09-29

**Authors:** Yazan Al Thaher, Hadil Faris Alotaibi, Lirong Yang, Polina Prokopovich

**Affiliations:** 1 School of Pharmacy and Pharmaceutical Sciences, Cardiff University, Cardiff, United Kingdom; 2 Department of Pharmaceutical Sciences, Pharmacy College, Princess Nourah bint Abdulrahman University, Riyadh, Saudi Arabia; Hamadan University of Medical Sciences, ISLAMIC REPUBLIC OF IRAN

## Abstract

Prosthetic joint infections (PJI) are still an extremely concerning eventuality after joint replacement surgery; growing antibiotic resistance is also limiting the prophylactic and treatment options. Chlorhexidine (a widely used topical non-antibiotic antimicrobial compound) coatings on silica nanoparticles capable of prolonged drug release have been successfully developed and characterised. Such nanocarriers were incorporated into commercial formulation PMMA bone cement (Cemex), without adversely affecting the mechanical performance. Moreover, the bone cement containing the developed nanocarriers showed superior antimicrobial activity against different bacterial species encountered in PJI, including clinical isolates already resistant to gentamicin. Cytocompatibility tests also showed non inferior performance of the bone cements containing chlorhexidine releasing silica nanocarriers to the equivalent commercial formulation.

## Introduction

Joint replacement surgery (arthroplasty) is generally performed for end-stage arthritis; the prevalence of such procedures is growing because of the continuous increase in obesity and aging population [1]. Prosthetic joint infection (PJI) is a possible consequence of arthroplasty observed in one to two percent of patients following primary total hip arthroplasty (THA) [2]. Unlike other types of post-operatory infection, PJIs can occur even months and years following surgery; nevertheless about a third of PJIs is diagnosed in the first months after arthroplasty [3] and about two third in the first year [4]. Different pathogens are responsible for PJI including: coagulase-negative *Staphylococci* (30–43%) and *Staphylococcus aureus* (12–23%), *Streptococci* (9–10%), Gram-negative bacilli (i.e. *Pseudomonas aeruginosa* and *Escherichia coli*) (3–6%), *Enterococci* (3–7%), and anaerobes (2–4%) [5]. Polymicrobial infections are diagnosed in 10–12% of cases [6,7]. The risks posed by PJIs to patients involve severe pain, poor health outcomes and possibly death with negative impact on patients’ quality of life [8]. Furthermore, PJIs are also a considerable economic burden to health providers as their management can require longer hospital admissions and repeated surgeries [9].

The use of antibiotics is the traditional approach for the prevention and treatment of PJIs [10] with gentamicin and tobramycin commonly mixed in PMMA bone cement in light of their broad spectrum activity and ability to withstand the high temperatures experienced by the settling bone cement dough. However, the continuing emergence of resistant microbial strains decreases the effectiveness of antibiotic-based therapies [11]. For example, 41% and 66% of *Staphylococci* isolated from PJI were resistant to gentamicin and tobramycin, respectively [12]. Moreover, antibiotic susceptibility of pathogens isolated in patients exposed to antibiotic laden bone cements (ALBC) was generally higher than in ALBC naïve, indicating the risk of selecting aminoglycosides resistant strains after using such materials [13]. Hence, it is extremely urgent to develop non-antibiotic therapies for the prevention and treatment of infections.

Chlorhexidine is a broad spectrum cationic bactericidal non antibiotic antimicrobial agent [14] widely used as a disinfectant and antiseptic for topical infections, cleaning wounds [15], sterilization of surgical instruments [16], and many dental applications including treatment of dental plaque, gingivitis and endodontic disease [17]. Despite its wide range of applications, it has not yet been broadly investigated in acrylic bone cements.

The available technologies to manage PJI are not completely satisfactory as infections are still not prevented and the reliance on antibiotic is likely to not sustainable long term because of antibiotic resistance. Antibiotic release from ALBC is completed after about 1 week for implantation so no protection is offered against infections developing later than this. The main unmet needs for PJI management are the provision of antimicrobial activity for periods of time of weeks and months and the use of non-antibiotic drugs. Any technology developed to address the occurrence of PJIs needs to fulfil specific requirements such as being able to resist the heat generated resulting from PMMA polymerisation, and assure that all bone cement material properties remain in the range mandated by regulators. Silica nanocarriers loaded with multilayer coatings containing gentamicin have shown to enable extended antimicrobial activity of PMMA bone cement without negative impact on other material properties [18,19]. We hypothesised that chlorhexidine could be embedded in multilayers coated silica nanocarriers in a similar approach and that, once mixed in bone cements, these nanoparticles could simultaneously address the challenge of PJI and antibiotic resistance. We assumed that the electrostatic charges exhibited by chlorhexidine would allow the deposition of this molecule in multilayers coatings on silica nanoparticles and drug release from the coatings would be sustained for prolonged periods of time. We further hypothesised that PMMA bone cement containing these silica nanocarriers would retain satisfactory material properties simultaneously enabling antimicrobial activity for longer periods of time than materials containing the same amount of free chlorhexidine.

## Materials and methods

### Chemicals

Triton X-100, tetraethyl orthosilicate (TEOS), 3-aminopropyltriethoxysilane (APTS), 1,6 hexanediol diacrylate, piperazine, sodium alginate, chlorhexidine diacetate, sodium acetate trihydrate, phosphate buffer solution (PBS) tablets, glutaraldehyde, Alizarin Red S, Calcein-AM, propidium iodide and “In Vitro Toxicology Assay Kit- LDH based” were purchased from Sigma-Aldrich, UK.

Cyclohexane, n-hexanol, ammonium hydroxide (35%), dichloromethane (DCM), diethyl ether, dimethyl-sulfoxide (DMSO), ethanol, methanol, glacial acetic acid and HPLC-grade acetonitrile were purchased from Fishers scientific, UK. Trihydrochloride Hoechst 33342 (Thermo Fisher Scientific, Eugene, OR, USA).

Cemex^®^ (Tecres^®^ SpA, Verona, Italy) bone cement was stored and used as from manufacturer’s guidelines.

Colorimetric ALP assay kit, picro-sirius red stain solution (ab150681), rabbit polyclonal antibodies against collagen I (ab21286), rabbit polyclonal antibodies against osteopontin (ab8448), rabbit polyclonal antibodies against osteocalcin (ab198228) and Alexa Fluor 488-labelled goat anti-rabbit IgG (ab150077) were purchased from Abcam, Cambridge, UK.

All reagents were stored according to manufacturer’s guidelines and used as received.

### Nanoparticle preparation and characterisation

#### Nanoparticle preparation amino functionalised silica nanoparticle synthesis

Silica nanoparticles functionalised with amine groups (SiO_2_-NH_2_) were prepared in one-pot synthesis by hydrolysis of TEOS in reverse micro-emulsion and subsequent functionalization with amino group (Stöber method) [20]. In a typical synthesis, Triton X-100 (17.7 g) was mixed with 16 mL of n-hexanol, 75 mL of cyclohexane, and 4.8 mL of deionised water under vigorous stirring. Once the solution was transparent, 600 μL of ammonium hydroxide (29.6%) was added. After sealing, the solution was stirred for 20 minutes and then 1 mL of TEOS was added; stirring continued for further 24 hours. The silica nanoparticles surface was functionalised with amino groups by adding 50 μL of APTS to the micro-emulsion under stirring and incubating further 24 hours. The SiO_2_-NH_2_ nanoparticles were recovered by adding 200 mL of ethanol and centrifuging at 14,000 rpm for 10 minutes (LE-80K, Ultracentrifuge, Beckman Coulter, UK) at 20°C (35,280 g). The nanoparticles were vigorously washed three times with deionized water and dry at room temperature in a fume hood for 24 hours.

#### PBAE synthesis

The synthesis of amino-terminated poly-β-amino esters (PBAEs) was carried out mixing 3.7 mmol of 1,6 hexanediol diacrylate with a 4.1 mmol of piperazine in 5 mL of DCM. The polymerization reaction was performed under stirring at 50°C for 48 h. The resulting amino terminated PBAE was precipitated in about 50 mL of diethyl ether followed by solvent evaporation under vacuum [18,19].

The amino functionalised silica nanoparticles were layered with different numbers of a repeating sequence of polyelectrolytes: sodium alginate, PBAE and chlorhexidine. The sequence of four layers (quadruple layer (QL)) alginate/chlorhexidine/alginate/PBAE constituted the minimal repeating unit. Up to ten quadruple layers were coated onto silica nanoparticles. The following concentrations of the polyelectrolytes and the drugs in acetic acid-sodium acetate buffer (pH 5) were used: sodium alginate (2 mg/mL), chlorhexidine (10 mg/mL) and PBAE (2 mg/mL) [18,19].

Dried amino functionalised silica nanoparticles were dispersed in 20 mL of sodium alginate solution and stirred for 10 min. The nanoparticles suspension was centrifuged, the supernatant replaced with 20 mL of acetate buffer, the nanoparticles resuspended and stirred for 10 min. The deposition of the first layer was completed with a further centrifugation and supernatant removal [18,19]. This process was repeated starting with resuspending the nanoparticles in the solution of the next polyelectrolyte in the sequence of layers. The coating was repeated up to the formation of 10 quadruple layers on the surface of the silica nanoparticles.

#### Nanoparticle characterisation

The size of nanoparticles was characterized using transmission electron microscopy (TEM). A droplet (4 μL) of nanoparticles suspension was placed on a plain carbon-coated copper TEM grid and left to evaporate in air under ambient laboratory conditions for few hours. Bright field TEM images at a magnification of 100,000 X were taken using a JEOL-1010 microscope at 80 kV equipped with a Gatan digital camera. Images were analysed with ImageJ software to measure the diameters of at least 150 particles [18,19].

Fourier-transformed infrared (FTIR) spectra (between 4,000 and 400 cm^−1^ with resolution of 4 cm^−1^) were recorded at room temperature on a Shimadzu IRAffinity-1S equipped with ATR diamond. A total of 16 scans per sample were averaged.

Thermogravimetric analysis (TGA) was performed using a Perkin-Elmer TGA 4000 instrument. The samples were heated from 50 to 800°C with a constant heating rate of 10°C/min. Sample weight was recorded and weight loss percentage of each sample was calculated relative to initial weight of sample, prior to heating. The organic material content of the nanocarriers was calculated by subtracting the weight loss at plateau (~ 750°C) to the weight loss at 100°C the is generally attributed to the water content of the nanoparticles [21].

Chlorhexidine release from the nanoparticles was evaluated by dispersing the drug loaded nanoparticles (10 mg) in an Eppendorf with 1 mL of acetate buffer pH 5 or PBS (pH 7.3). Samples were vigorously stirred in a vortex and then incubated at 37°C; every 24 hours the release medium was removed for further chlorhexidine quantification and replaced with 1 mL of fresh buffer [18,19].

### Chlorhexidine quantification

Chlorhexidine concentration in the buffer solutions was quantified using reversed-phase High Performance Liquid Chromatography (rp-HPLC). An HPLC system (1100 series Agilent Technologies^®^) equipped with a μBondapak C18 column (pore size 125 Aº, 10 μm, 3.9 mm X 150 mm) purchased from Waters^®^, Ireland and a UV detector at 239 nm was employed. 20 μL of sample were injected in a mobile phase made of a mixture of acetate buffer pH 4 and acetonitrile (73:27) at a constant flow rate of 1 mL/min. Standards of known chlorhexidine concentrations ranging 250 to 1 mg/L were analysed to establish a calibration curve and detection limit.

### Bone cement preparation and characterization

#### Bone cement preparation

Bone cement preparation was carried out according to manufacturer’s instructions and the ISO5833:2002 (Implants for surgery-Acrylic resin cements). All the contents of the bone cement were stored at recommended conditions (20–25 ºC for the powder and 8–15 ºC for the liquid in the dark) and conditioned to room temperature (22 ºC) for 2 hours before mixing.

Chlorhexidine powder or the silica coated nanoparticles containing an equivalent amount of drug was added to the solid phase of bone cement and carefully mixed.

Both components were weighted and hand mixed in a polypropylene bowl with a polypropylene spatula for 1 min and then poured into a polytetrafluoroethylene (PTFE) mould with specific dimensions and shapes according to the chosen tests. After pouring the cement dough, the mould was clamped with two steel endplates covered with PTFE films on both sides. After 2 hours, the samples were pushed out of the mould and allowed to cure for 24±2 hours at 23 ºC. The samples were sanded down to smooth the edges using 320 grit silicon carbide paper.

#### Rheology testing

The effect of adding the nanoparticles on the cement settling time was evaluated through rheological tests using MRC702 (Anton Paar Ltd., UK), equipped with 6 mm diameter circular flat plates.

Test were conducted at room temperature, plate distance 1 mm, a strain of 0.1% and fixed frequency of 1 rad/sec. In all tests, the bone cement solid phase was mixed with the liquid phase quickly with a spatula; the mixture was deposited onto the lower plate, the top plate was lowered into position and then experiments started. To account for the time elapsed during mixing and pouring in each analysis, the time recorded during the rheological test was offset by the manually measured time intervened between start mixing the two PMMA bone cement components and the start of the rheological measurements. The setting time was extracted from each curve as the time corresponding to a local maximum of *tan δ* (where *δ* = G"/G’). Each experiment was carried out on three independently prepared cement samples, and results are presented as mean and standard deviation [19].

#### Drug release quantification

Bone cement cylindrical samples with 6 mm diameter and 10 mm length were prepared for these tests. Samples weighed 0.40 ± 0.01 g and were incubated in 3 mL PBS buffer (pH 7) at 37 ºC. The release media was replaced each day to attain sink condition, where the concentration of released drug is negligible in comparison to its saturation limit [18,19]. Three samples from three independent batches of nanoparticles were used for each type of bone cement.

#### Antimicrobial testing

Gram-positive bacteria methicillin-resistant *Staphylococcus aureus* (MRSA) NCTC12493, *Streptococcus pyogenes* ATCC19615 and *Staphylococcus epidermidis* ATCC12228 along with Gram-negative bacterium *Acinetobacter baumannii* NCIMB9214, *Pseudomonas aeruginosa* NCIMB10548, *Escherichia coli* NCTC10418 were used. Gentamicin resistant PJI clinical isolated strain were also tested: MRSA 275 and 294; *S*. *epidermidis* 272, 222 and 199; Methicillin resistant *S*. *epidermidis* (MRSE) 140, *A*. *baumannii* 646, 643, 640; *E*. *coli* 293 and *Enterococcus* 181. Bacteria frozen stokes were stored at -80°C; strains were streaked on BHI plates weekly and incubated for 18–24 hours at 37°C, then stored at 4°C.

Bacteria were inoculated in BHI broth and incubated for 24 hours at 37 ºC; cell suspensions were diluted 1:1000 in fresh sterile BHI broth to get bacterial count in the range of 10^4^−10^5^ CFU/mL. 20 μL of the diluted broth were added into a sterile 96-well plate. After that, each well was filled with 100 μl of the release media of different types of bone cement, and the plate was incubated for 24 hours at 37 ºC. The growth in each well was evaluated visually the following day. Each data point was performed on three independent cultures of all individual strains on 6 individual batches of bone cements specimens; the duration of the inhibitory activity towards bacteria of the bone cement was determined as the day corresponding to the last daily release media inhibiting bacterial growth [18,19].

#### Material testing

Test were conducted with Zwick Roell ProLine table-top Z050/Z100 equipped with a dedicated software package (TestXpert II software, Zwick Testing Machines, Herefordshire, UK).

Compression tests were conducted in accordance international guidelines [22] using cylindrical shaped specimens (diameter = 6 mm and height = 12 mm) at a constant crosshead speed of 20 mm/min. Compressive strength was determined both before and after the test specimens were incubated at 37°C in PBS for 90 days.

Bending strength and bending modulus were determined through four-point bending tests [22] using rectangular specimens (length = 75 mm, width = 10 mm and thickness = 3.3 mm) at a cross-head displacement rate of 5 mm/min.

Fracture toughness tests were conducted using rectangular specimens (length = 45, width = 10 and thickness 3.3 mm) were a sharp chevron notch (5.5 ± 0.5 mm deep) was cut into the centre of one of the long sides of the specimen using a sharp razor blade [22]. The specimens were loaded at the centre of the unnotched long face, in three-point bend mode (distance between support rollers = 40 mm), at a cross-head displacement rate of 5 mm/min.

6 specimens were tested for each combination of cement and mechanical test, moreover when the bone cements were mixed with nanoparticles these were independent batches.

Bone cement samples were incubated in 3 mL PBS at 37°C for 90 days; for the first 14 days, the samples were weighed daily; after that the samples were weighed every 3 days [18,19]. Water uptake was calculated by dividing the increase in sample weight at different time points by the sample weight at time zero.

#### Cytotoxicity testing

Saos-2 human osteosarcoma osteoblast-like cells (ATCC^®^ HTB-85^™^) were cultured in RPMI-1640 medium supplemented with foetal bovine serum (10% v/v) and 1% v/v of penicillin (5000 U/mL)/streptomycin (5000 mg/mL). Cells were incubated at 37°C in humidified atmosphere with 5% CO_2_.

Bone cement samples (cylinder shaped with length of 12 mm and diameter = 6 mm) were incubated in growth medium for 24 hours at 37°C to obtained growth medium containing compounds released from the bone cements (denoted as “bone cements release medium” from here onward).

Saos-2 cells were seeded on glass coverslips (diameter 16 mm) deposited in 6-well plates at a density of 7.2x10^4^ cells/well (counted using Trypan Blue to differentiate between viable and nonviable cells) and incubated for 24 hours to allow cell attachment. Growth medium was replaced with bone cements release medium and changed twice per week.

MTT test was carried out after 1, 2, 4 and 7 days of incubation replacing the media with 1 ml of phenol red-free medium containing 5 mg/mL of MTT reagent, the plates were incubated for 2 hours at 37°C in humidified atmosphere with 5% CO_2_. Medium was removed, 150 μl of DMSO was added to each well and the plates were incubated for further 10 min. The solution containing the dissolved formazan was analysed with a spectrophotometer (Tecan^®^ Infinite F50, Austria) at 560 nm.

LDH was quantified in the media (LDH_released_) and after adding the cell lysis solution (LDH_total_) after 1 day, 2 days, 4 days and 7 days of incubation according to manufacturer’s protocols. Total and released were determined as OD, at 490 nm, after correcting for the reading from the negative control. Cell viability was calculated according to the following equation [23]:

$$viability\;(\%)=\frac{{LDH}_{total}-{LDH}_{released}}{{LDH}_{total}}*100$$ (1)

#### Histology

Cells cultured in bone cements release medium for 2 days, were washed three times with PBS. 3 ml of PBS containing 0.1% w/v propidium iodide, 0.2% w/v calcein-AM and 5 μg/mL Hoechst were added to each well and the cells were incubated at 37°C for 30 min; cells were washed with PBS immediately before imaging with confocal microscopy (Zeiss, Oberkochen, Germany).

*1*.*1*.*1*.*1 Actin filaments staining*. Cells cultured in bone cements release medium for 2 days, were washed three times with PBS, fixed with 4% paraformaldehyde in PBS pH 7.4 for 30 min at room temperature and permeabilised with 0.1% Triton-X-100. 3 ml of PBS containing 50 μg/ml fluorescent phalloidin conjugate and 5 μg/mL Hoechst were added in each well and plates incubated at 37°C for 30 min; cells were washed with PBS immediately before imaging with a confocal microscope (Zeiss, Oberkochen, Germany).

Cells cultured in bone cements release medium for 21 days were washed three times with PBS then fixed using 4% paraformaldehyde in PBS pH 7.4 for 30 min at room temperature. After washing three times with distilled water, cells were stained with 1% (w/v) Alizarin Red S for 30 min at room temperature with shaking to evaluate calcium deposits in cells. Cells were washed several times with distilled water and 800 μL of 10% acetic acid was added to each well containing stained cells on glass coverslips in new 6-well plates and incubated for 30 min at room temperature with shaking. Cell lysates were collected in Eppendorf tubes and vortexed for 30 seconds. After heating at 85°C for 10 min, Eppendorf tubes were placed on ice for 5 minutes and then centrifuged at 12,000 rpm for 15 min. 200 μL of the supernatant was transferred to a 96-well plate with flat bottom and measured at 405 nm in a multi-well microplate reader (LT-5000MS ELISA reader, Labtech).

Cells cultured in bone cements release medium for 7 days, were washed three times with PBS and fixed with 4% paraformaldehyde in PBS pH 7.4 for 30 min at room temperature. Then cells were stained with 1-stepTM nitro blue tetrazolium (NBT)/5-bromo-4-chloro-3-indolyl phosphate (BCIP) (Thermo scientific, Rockford, IL, USA), which was a one-component sensitive precipitating alkaline phosphatase substrate. Cells were washed three times with PBS then lysed with 300 μL/well pierce radioimmunoprecipitation assay (RIPA) lysis and extraction buffer (Thermo Scientific, Rockford, IL, USA). The plates were kept on ice for 15 min with occasionally swirling. Cell lysates were collected Eppendorf tubes and centrifuged at 12,000 rpm for 15 min. The supernatant was transferred to a new tube as samples and analysed by a colorimetric ALP assay kit according to the manufacturer’s instruction. The coloured p-nitrophenol resulting from the conversion of pNPP by ALP was quantified at 405 nm in a multiwell microplate reader (LT-5000MS ELISA reader, Labtech).

Cells cultured in bone cements release medium for 7 days, were washed three times with PBS and the collagen secreted was stained with 1 ml/well picro-sirius red stain solution and incubate at room temperature for one hour. After rinsing quickly 2 times with acetic acid solution, the stained cells were rinsed 2 times with distilled water and dissolved in a solution containing equal volume of methanol and 0.2M NaOH. The coloured solution was transferred to a 96-well plate with flat bottom and analysed at 560 nm in a multiwell microplate reader (LT-5000MS ELISA reader, Labtech). Each sample was triplicate.

Immunofluorescent staining was conducted to evaluate osteogenic marker proteins expressed in cells, such as COLI, OPN and OCN. Cells cultured in bone cements release medium for 7 days (for COLI and OPN) or 14 days (for OCN), were washed three times with PBS then fixed using 4% paraformaldehyde in PBS pH 7.4 for 30 min at room temperature. After washing three times with PBS, cells were permeabilised using 0.1% Triton X-100 for another 10 min at room temperature. Cells were again washed three times with PBS and incubated with 10% goat serum for 30 min at room temperature to block unspecific binding of the antibodies to reduce background staining. After this, cells were incubated in the 100 times diluted first antibody (such as rabbit polyclonal antibodies against collagen I, rabbit polyclonal antibodies against osteopontin and rabbit polyclonal antibodies against osteocalcin) in 10% goat serum for 1 hour at room temperature. After washing three times with PBS, cells were incubated in the 500 times diluted second antibody, Alexa Fluor 488-labelled goat anti-rabbit IgG in 10% goat serum for 1 hour at room temperature in the dark. Cell nuclei were counterstained with trihydrochloride Hoechst for 10 min at room temperature. Images were acquired using a laser scanning confocal microscope (Zeiss, Oberkochen, Germany).

### Statistical analysis

All data were expressed as means ± standard deviation (SD) from at least three independent values.

Normal distribution of the nanoparticles size was assessed with the Shapiro-Wilk test and quantile-quantile (Q-Q) plots; to assess the statistical significance of results between groups, one-way analysis of variance (ANOVA) was performed. Multivariate analysis of variance (MANOVA) was carried out using the Pillai test to assess the antimicrobial activity of the nanocarriers against the pure chlorhexidine powder considering the number of days the growth was prevented as multiple dependent variables. Experimental results were considered statistically significant at 95% confidence level (*p*<0.05). All analyses were run using the R (ver 4.0) [24].

## Results

### Nanoparticle characterization

**Fig 1:**
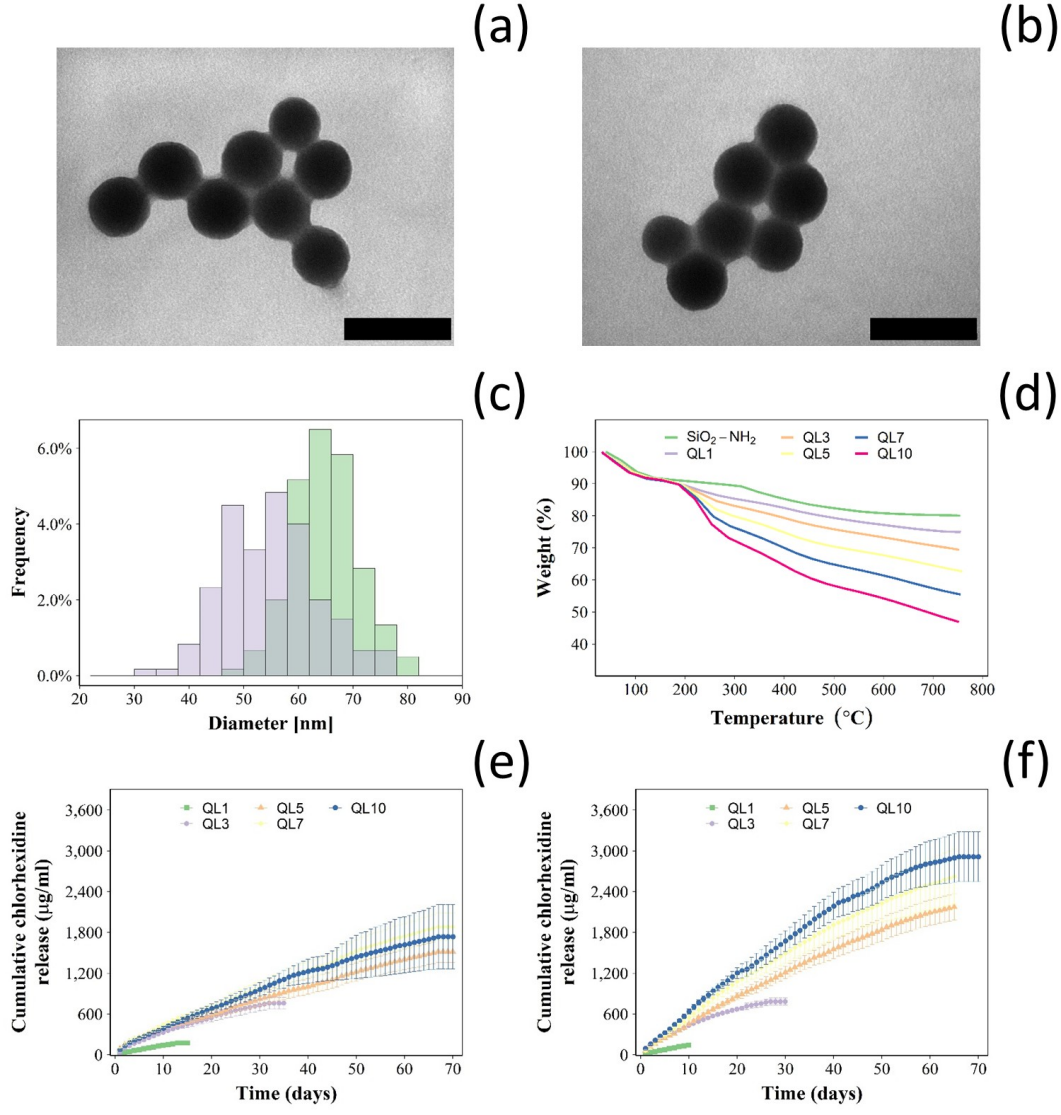
Characterisation of silica nanocarriers. Example of TEM images (bar represents 100 nm) of (a) amino functionalised silica nanoparticles and (b) after deposition of 10 QL. Size distribution of amino functionalised silica nanoparticles (violet) and after the deposition of 10 QL (green) (c); examples of thermogram for silica nanoparticles layered with different number of quadruple layer containing chlorhexidine (d). Cumulative chlorhexidine release for silica nanoparticles layered with different number of quadruple layer in buffer pH 5 (e) and pH 7.3 (f) (mean ± SD, n = 3).

The synthesised nanoparticles were rounded (Fig 1a and 1b); particles diameters were normally distributed (Fig 1c and S1 Fig) with a mean ± SD of 55.1 ± 8.3 nm and 66.2 ± 6.2 nm for the uncoated silica NPs and after the deposition of 10 QL, respectively.

Thermograms of the synthesised nanoparticles exhibited a weight drop at around 100°C and a further weight loss starting at around 200°C reaching a plateau when the temperature was ~ 650°C (Fig 1d). The organic content (Table 1) of the uncoated amino functionalised nanoparticles was 14.95% and increased with growing numbers of deposited QL reaching ~50% after 10 QL were layered in the nanoparticles.

**Table 1:** Percentage of organic matter in silica nanoparticles layered with different number of quadruple layers containing chlorhexidine determined from TGA (n = 3 ± SD).

Nanoparticles	Organic content (%)
**SiNH_2_**	14.95 ± 1.00
**QL1**	20.18 ± 1.51
**QL3**	25.95 ± 0.36
**QL5**	33.08 ± 2.14
**QL7**	40.45 ± 1.45
**QL10**	49.59 ± 2.25

Chlorhexidine release kinetics and overall duration from the coated nanoparticles immersed in aqueous buffers increased with the number of deposited multilayers, up to 70 days (Fig 1e and 1f). At pH 5, the same trend in the increase in the release kinetics and duration with increasing the number of quadruple layers was also observed. However, for comparable number of deposited layers, realise kinetic and total amount of drug released were lower at pH = 5 than pH = 7.3. In both conditions, the release had an exponential decay profile with the greater release at t = 0 progressively decreasing down to zero.

**Fig 2:**
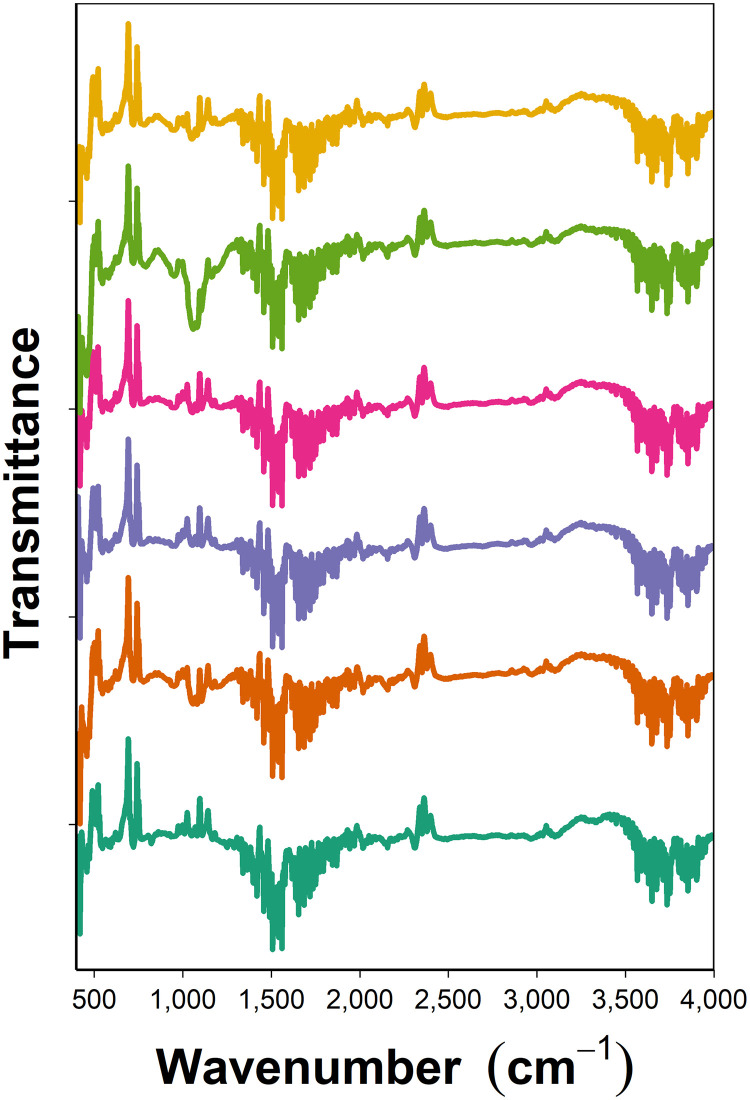
FTIR of chlorhexidine coated nanocarriers. Example of FTIR spectra of pure chlorhexidine (**—**) and of the coated nanocarriers after deposition of chlorhexidine during construction of QL1 (**—**), QL3 (**—**), QL5 (**—**), QL7 (**—**) and QL10 (**—**).

FTIR demonstrated the efficacy of the coating process as the outer surface of the nanocarriers exhibited the same spectra as the pure compound deposited (Fig 2).

### Bone cement

#### Chlorhexidine release profile and antimicrobial activity

As no commercial bone cement containing chlorhexidine is available, the addition of different amounts of pure chlorhexidine was studied to determine the suitable concentration of this antimicrobial agent.

**Fig 3:**
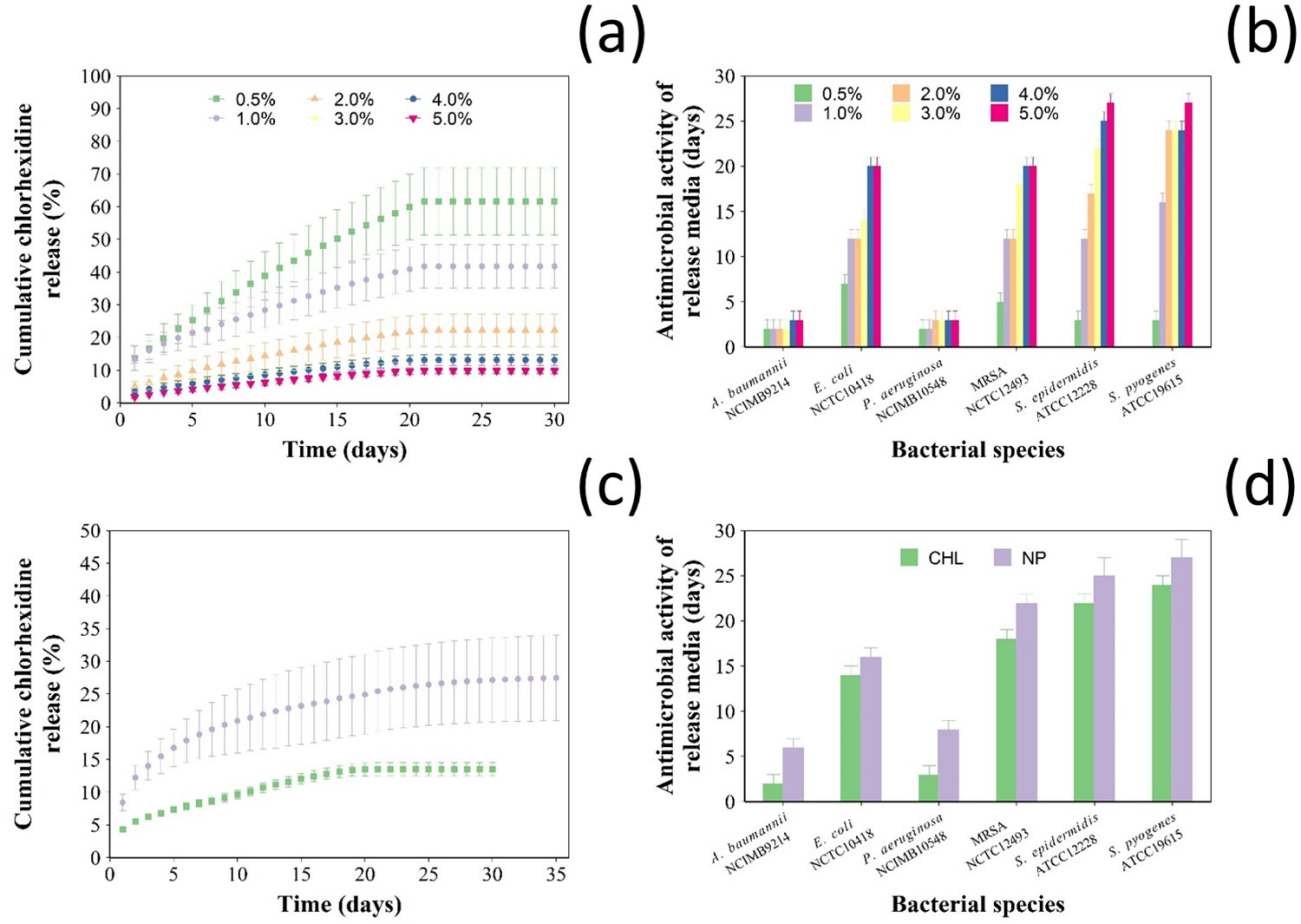
Chlorhexidine release from nanocarriers. (a) Cumulative chlorhexidine release in PBS (pH 7.3) and (b) duration of antimicrobial activity of Cemex^®^ with different concentrations of pure chlorhexidine (0.5, 1, 2, 3, 4, 5% w/w) (mean ± SD, n = 3). (c) Cumulative percentage of chlorhexidine release in PBS (pH 7.3) and (d) duration of antimicrobial activity of Cemex with 3% w/w chlorhexidine either as pure powder or in coated NPs (mean ± SE, n = 6).

Release of chlorhexidine from bone cement samples was not detected after 22 days regardless the initial concentration of drug added to the bone cement (Fig 3a). Not all the chlorhexidine added to the bone cement dough was released from the bone cement samples; the proportion of drug reaming entrapped in the samples was directly correlated to the initial amount.

There was no significant difference in the antimicrobial activity for the different bone cements (0.5–5% w/w) against *A*. *baumannii* and *P*. *aeruginosa*; the inhibition lasted less than 4 days (Fig 3b). The antimicrobial activity of Cemex containing 3, 4 and 5% w/w of pure chlorhexidine was around 20 days against *MRSA* and *E*. *coli* and around 25 days for *S*. *pyogenes* and *S*. *epidermidis*. As a result, the addition of 3% w/w of pure chlorhexidine was chosen for further optimization, because it seemed that higher concentrations of chlorhexidine (4 and 5%) did not significantly increase in the antimicrobial activity and higher amounts may compromise the mechanical properties of the cement or increase toxicity without improving the antimicrobial properties.

When 3% (w/w) of chlorhexidine was added to Cemex through the coated silica nanoparticles, drug release continued for up to 30 days in the nanocomposite and 22 days for pure chlorhexidine (Fig 3c). In terms of cumulative release, 33% of the initial chlorhexidine present in the bone cement samples was released when coated silica nanocarriers were used; this was about twice that of pure chlorhexidine (16% released).

Cemex containing chlorhexidine loaded nanoparticles showed longer duration of bacterial growth inhibition (Fig 3d) compared to bone cement containing the same amount of antimicrobial drug against *A*. *baumannii* and *P*. *aeruginosa* for 6 and 8 days, respectively, compared to 2 and 3 days (p < 0.05). Cemex loaded with nanoparticles inhibited the growth for up to 25 days for *S*. *epidermidis* and 22 days for *MRSA*, compared to 22 and 18 days respectively for pure chlorhexidine. Also, there was no significant difference in the antimicrobial activity of Cemex powder or nanoparticle containing bone cement against *S*. *pyogenes* and *E*. *coli* (p > 0.05).

**Fig 4:**
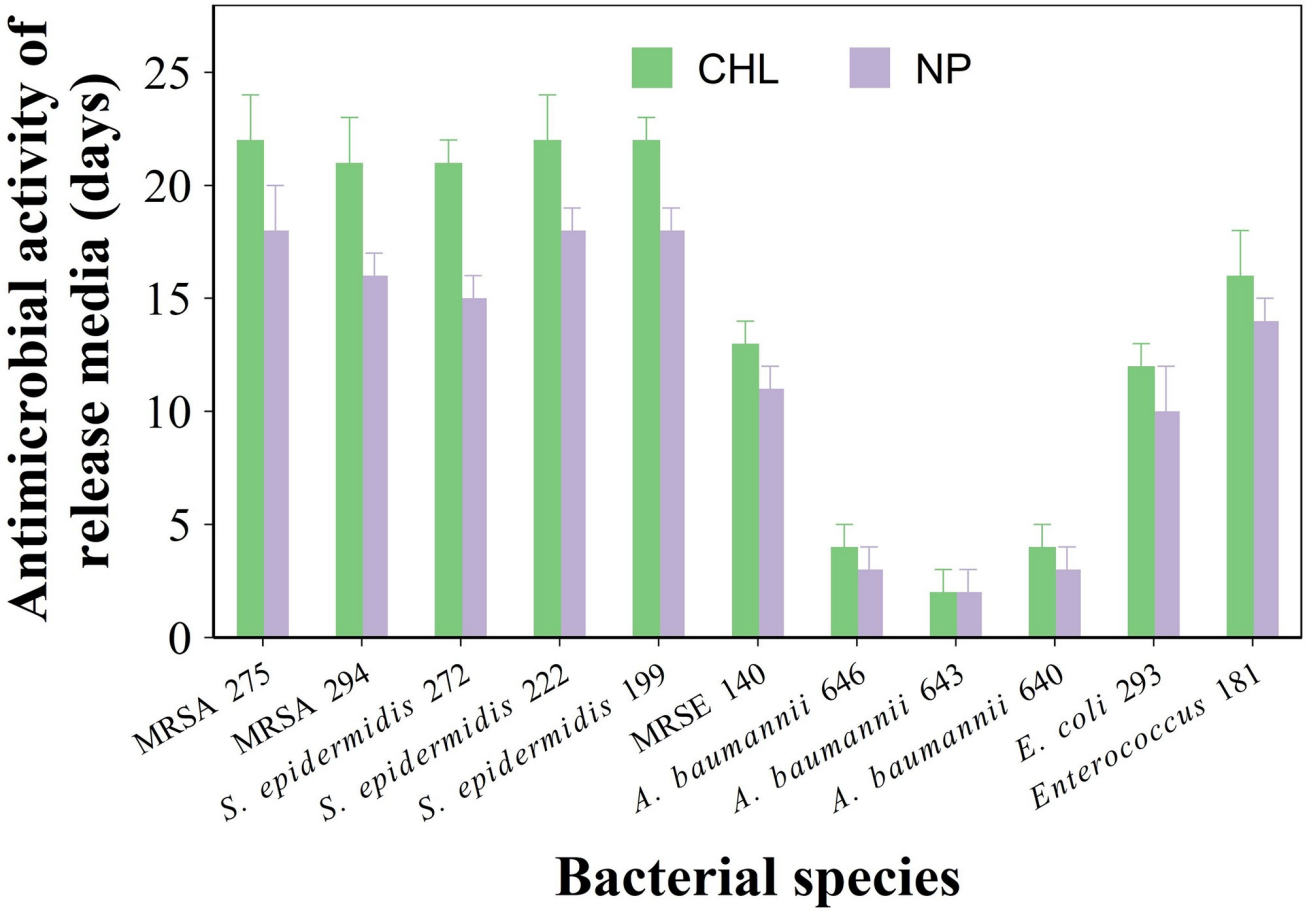
Antimicrobial activity of bone cement. Duration of antimicrobial activity against gentamicin resistant clinical isolates of PJI of Cemex with 3% w/w chlorhexidine either as pure powder or in coated NPs (mean ± SE, n = 6).

The ability of the sample containing the developed nanocarriers to prevent PJI clinical isolates growth had a similar pattern; Cemex containing chlorhexidine releasing nanocarriers had generally longer antimicrobial activity than Cemex with chlorhexidine in pure form (Fig 4).

#### Material properties

The possible effect of the chlorhexidine nanoparticles on the kinetics of different bone cements settling time was investigated through the evaluation of the rheological properties of bone cement dough after mixing.

**Fig 5:**
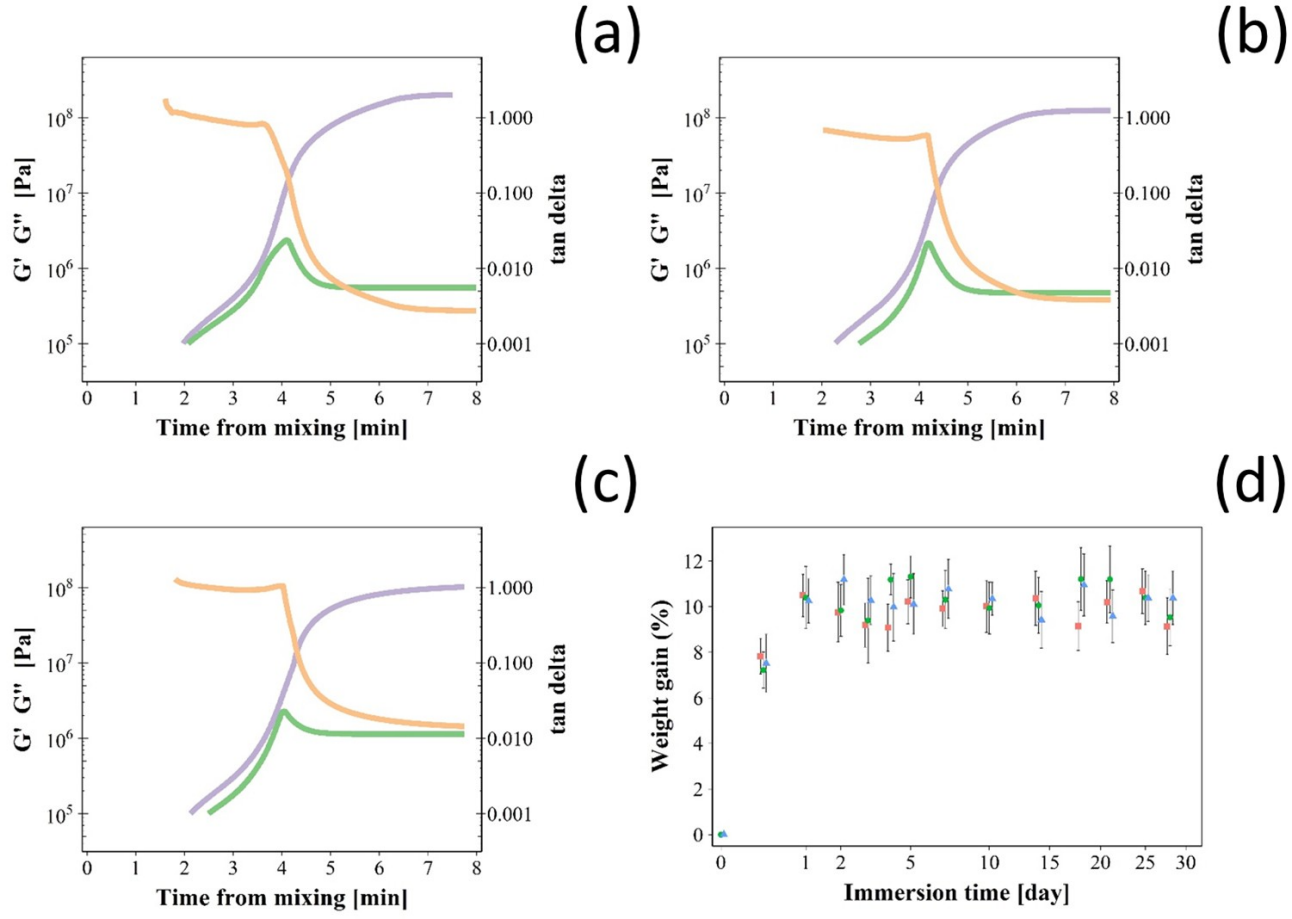
Bone cement settling. Time-dependent rheological measurements (tan δ in orange, storage modulus (G’) in violet and loss modulus (G”) in green) during curing for Cemex^®^ bone cement (a), Cemex^®^ containing chlorhexidine powder 3% w/w (b) and coated silica nanoparticles (c). (d) Water uptake for Cemex^®^ bone cement (green), Cemex^®^ containing chlorhexidine powder 3% w/w (violet) and coated silica nanoparticles (orange) during incubation in PBS buffer (mean ± SD, n = 3).

During curing, storage modulus (G′) and loss modulus (G″) increased initially after mixing the bone cement phases reaching a plateau (Fig 5a–5c); the ratio between the two described by *tan δ* exhibited a local maximum instead. This represented the time required by the bone cement dough to settle. The addition of the silica nanoparticles or pure chlorohexidine did not impact the settling time of Cemex bone cement as it was ~4 minutes in all cases tested (Table 2).

**Table 2:** Curing times in minutes (mean and standard deviation) Cemex^®^ bone cement, Cemex^®^ containing chlorhexidine 3% w/w as powder or through coated silica nanoparticles.

Bone cement	Curing time (min)
**Cemex** ^**®**^	4.1 ± 0.5
**Cemex** ^**®**^ **+ chlorhexidine powder 3% w/w**	4.3 ± 0.5
**Cemex** ^**®**^ **+ 3% w/w chlorhexidine in coated silica nanoparticles**	4.2 ± 0.7

Bone cement samples immersed in PBS absorbed liquid during the first few days of contact then the amount of fluid inside the samples remained constant for the rest of the observed period (Fig 5d); the differences in liquid uptake among the bone cements tested were not statistically significant (p > 0.05).

**Fig 6:**
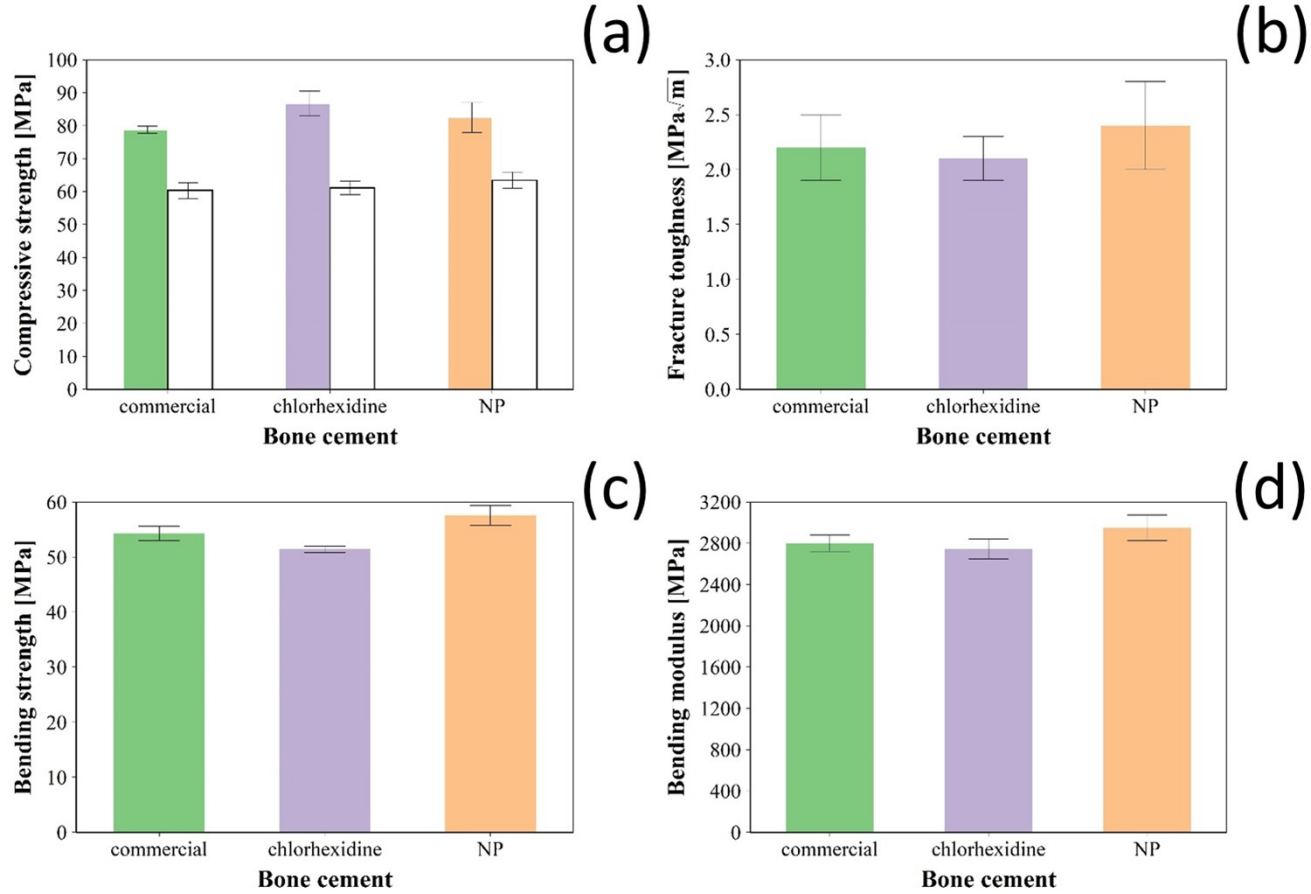
Mechanical properties of bone cement. (a) Compressive strength after settling (coloured bars) and after 3 months incubation in PBS (white bars); (b) fraction toughness, (c) bending strength and (d) bending modulus of Cemex^®^ bone cement (green), Cemex^®^ containing chlorhexidine powder 3% w/w (violet) and coated silica nanoparticles (orange) (mean ± SD, n = 6).

All types of bone cements tested had similar compressive strength (p > 0.05) (Fig 6a) either after settling or after incubation in PBS for 90 days; however, compressive strength of all samples decreased after incubation in liquid media (p < 0.001).

Bending strength and bending modulus (Fig 6c and 6d) for Cemex containing either chlorhexidine 3% w/w or an equivalent amount of drug in coated silica nanoparticles were similar to commercial cement Cemex (p > 0.05), and met the ISO requirements (> 50 MPa). In addition, the differences in fracture toughness (Fig 6b) of these bone cements were not statistically significant (p > 0.05).

#### Cytotoxicity analysis

**Fig 7:**
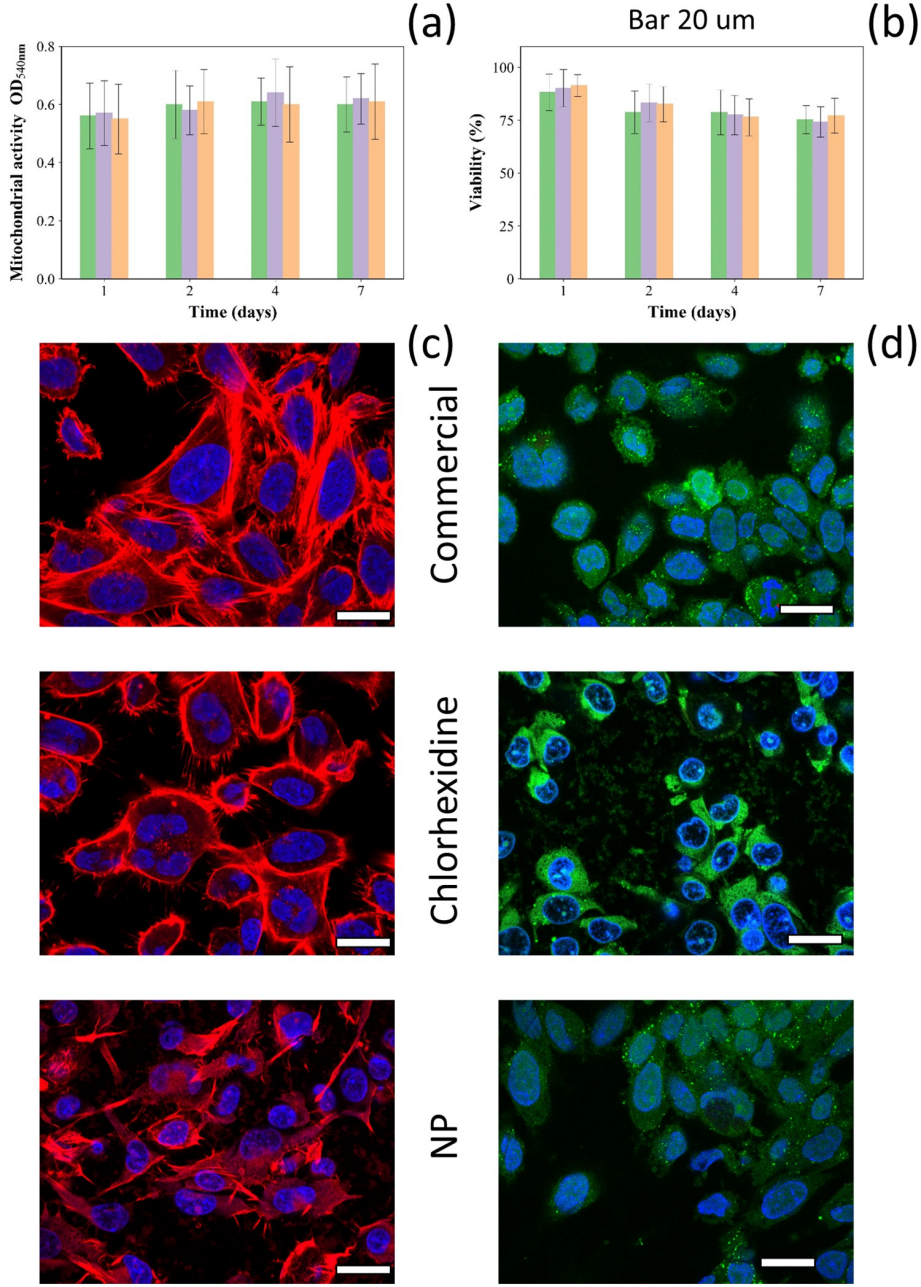
Cytocompatibility of bone cement. (a) Mitochondrial activity (MTT assay) and (b) Viability ratio (composite/commercial cement) of SaoS-2 cells exposed to Cemex^®^ bone cement (green), Cemex^®^ containing chlorhexidine powder 3% w/w (violet) and coated silica nanoparticles (orange) for different periods of time (mean ± SD, n = 6). (c) Actin/DAPI and (d) live/dead staining images of SaoS-2 cells exposed to release medium of different bone cement samples. (bar represents 20 μm).

Osteoblast cells exposed for up to 7 days to release media from Cemex (commercial formulation with no added antibiotic), Cemex containing 3% w/w chlorhexidine either as pure powder of through silica coated nanoparticles showed similar mitochondrial activity (MTT assay) and cell viability (LDH assay) (Fig 7a and 7b) (p > 0.05). Osteoblast cells exposed to the release medium of different types of bone cements showed similar viability (cells stained in green) and similar actin filaments (in red) spreading (Fig 7c and 7d).

**Fig 8:**
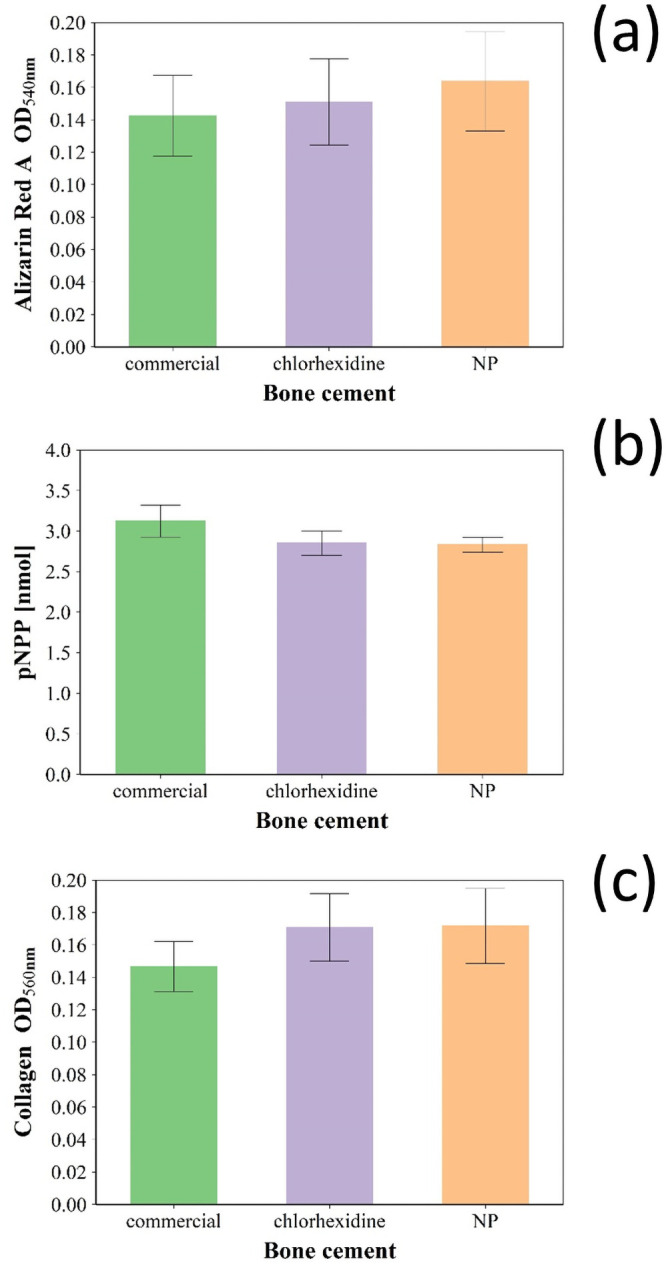
Evaluation of osteoblasts activity. (a) Quantification of the mineralisation of Saos-2 cells with alizarin red S after 21 days of exposure to release media. (b) ALP activity quantification in Saos-2 cells after 7 days of exposure to release media. (c) Collagen excreted by Saos-2 cells quantification after 7 days of exposure to release media. Cemex^®^ bone cement (green). Cemex^®^ containing chlorhexidine powder 3% w/w (violet) and coated silica nanoparticles (orange) (mean ± SD, n = 6).

Mineralisation ability, ALP activity and collagen secretion of Saos-2 cells exposed to release media from bone cement samples were assessed both qualitatively (microscopy) and quantitatively. The mineralisation ability of Saos-2 exposed to growth medium containing release compounds from bone cements was determined through alizarin red S staining (a dye binding calcium ions/deposits) and did not appear to be affected (p > 0.05) by the addition of chlorhexidine to commercial bone cement (Fig 8a). There was no significant difference (p > 0.05) in ALP activity between Saos-2 cells exposed for 7 days to release media of commercial bone cement or after the addition of either chlorhexidine or silica coated nanoparticles (Fig 8b). The presence in the bone cement sample of chlorhexidine (3% w/w) as pure powder form or in the coating of silica nanoparticles did not affect (p > 0.05) the level collagen secretion of Saos-2 cells cultured for 7 days in bone cement release media compared to commercial bone cement formulation (Fig 8c).

**Fig 9:**
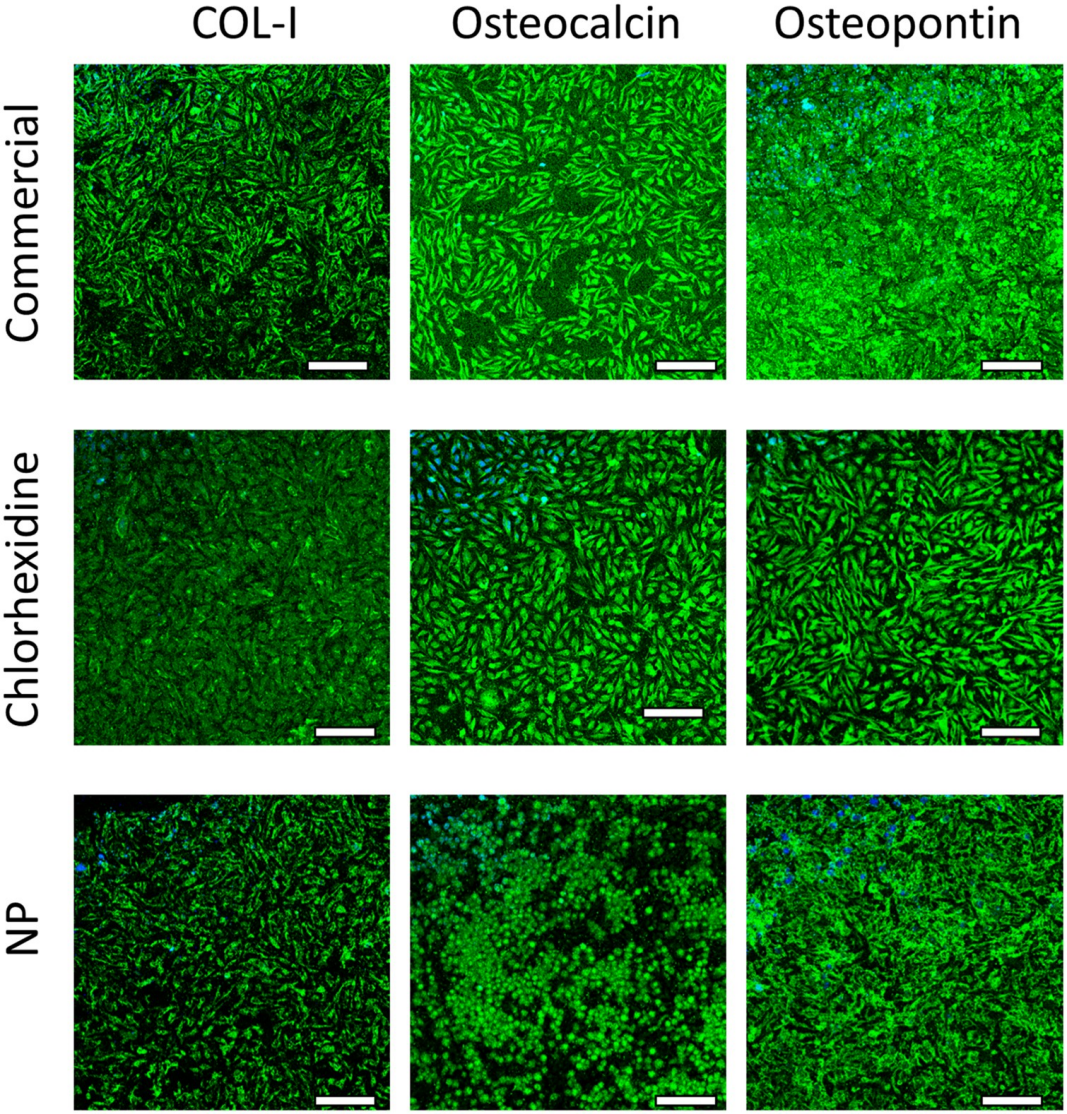
Histological testing of bone cement. Examples of fluorescence microscope images of collagen type1, osteopontin and osteocalcin in SaoS-2 cells after exposure to release media from Cemex^®^ bone cement (a), Cemex^®^ containing chlorhexidine powder 3% w/w (b) and coated silica nanoparticles (c) (bar represents 100 μm).

Immunofluorescent staining was employed to assess the expression of osteogenesis-related proteins (specifically type1 collagen, osteopontin and osteocalcin). Saos-2 cells exposed to release media from commercial bone cement displayed high intensity fluorescence for the three proteins; the addition of chlorhexidine to the bone cement did not appear to impact the expression of any of these proteins as no impact on fluorescence intensity was observable (Fig 9).

## Discussion

Multilayer coatings releasing chlorhexidine could provide a solution to the potentially life changing and life-threatening consequences of PJIs; furthermore this approach could also contribute to the fight against antibiotic resistance. The feasibility of the technology deploying multilayer coated silica nanoparticles was validated, first through characterisation of the nanoparticles and drug release properties of the nanocarriers, then a suitable amount of chlorhexidine to be added to PMMA bone cement was determined in order to allow comparison with materials containing an equivalent amount of drug in the coated nanoparticles. Finally, the suitability of PMMA bone cement containing the developed nanocarriers was assessed through a variety of mechanical, cytocompatibility and histological characterisation. The choice of chlorhexidine as antimicrobial agent was driven by the non-antibiotic nature of such molecule, its broad-spectrum antimicrobial activity, structure exhibiting positive charges to allow deposition and proven safety in numerous not orthopaedic applications.

The physico-chemical characterisation of the nanocarriers showed that the thermogram for the amino functionalised silica nanoparticles (Fig 1d) was similar to other previous reports [25]. Moreover, the organic content in the amino functionalised silica nanoparticles (Table 1) was in agreement with other reports [26]. The consistent increase in the organic content observed for increasing number of layers, along with FTIR spectra (Fig 2), confirmed deposition of polyelectrolytes and drug on the surface of the amino functionalised silica nanoparticles. Compared to nanoparticles covered with 10 quadruple layers of gentamicin releasing coatings (organic content 41.5 ± 3.6%), chlorhexidine loaded nanoparticles had higher organic matter of 49.5 ± 2.2% which was expected because chlorhexidine has higher molecular weight and higher positive charge than gentamicin; two factors favouring effective deposition.

*In vitro* release studies were performed to investigate the chlorhexidine release profile from both types of coatings at different joint conditions, healthy joint (pH ~ 7.3) and infected joint associated with local acidosis (pH ~ 5) [27,28]. Drug release from multilayers coatings was mainly dependent on the number of quadruple layers, type of polyelectrolytes, electrostatic interaction between polyelectrolytes and kinetics of hydrolysis for the polyelectrolytes involved [29–31]. Therefore, environmental pH affects the release kinetic influencing polyelectrolytes hydrolysis and protonation that subsequently controls drug diffusion through coating [32]. PBAE charge at pH 7 is close to zero and positive at pH 5 [32] resulting in weaker interactions, hence high drug diffusion, with alginate at neutral than acidic conditions; determining a chlorhexidine higher release kinetic at pH 7 compared to pH 5, a similar trend was observed with gentamicin release [18,19,32]. PBAE hydrolysis could be another possible mechanism involved in drug release but the degradation rate is slower than drug diffusion [32]; therefore delamination is not the controlling mechanism of chlorhexidine release from the developed multilayers coatings.

No commercial bone cement containing chlorhexidine is available; therefore a suitable amount of such drug to be added was selected based on the antimicrobial activity against a series of pathogens encountered in PJI. Cemex bone cement with 3% w/w of chlorhexidine provided similar antimicrobial properties as those of the Cemex with gentamicin routinely employed in clinical practice (standard of care) and thus this dose was selected in further comparative experiments. Bone cement containing chlorhexidine releasing silica nanocarriers had longer drug release profile (Fig 3c) and, consequently, longer antimicrobial activity (Fig 3d) than samples containing the same amount of free drug confirming the feasibility of the approach. Furthermore, encapsulation of chlorhexidine in multilayers coatings also resulted in a better release efficacy of the initial drug added to the bone cement dough. It is well known that only about a fifth of antibiotic added to PMMA bone cements is released, this has been attributed to drug entrapment in the bone cement matrix. However, more recent observations have also raised the possibility that the added drug could undergo a process of inactivation during bone cement setting and thus protection of the antimicrobial molecule, for example in liposomes or multilayers coatings, could results in a reduced drug loss and greater release yield [19,33] explaining the release profiles observed (Fig 3c).

The antimicrobial activity of the bone cement samples was due to the released drug; as the concentration of the antimicrobial agent in the media surrounding the bone cement decreases with time, PJI can only be prevented when the concentration is above the MIC of pathogens. The variations observed among the tested strains is linked to MIC towards a specific microorganism being a strain specific property; furthermore Gram- (i.e. *A*. *baumannii* and *P*. *aeruginosa*) are generally more resistant than Gram+ (i.e. *S*. *aureus*, *S*. *epidermidis* and *S*. *pyogenes*) to antimicrobial treatment and this was also observed from the bone cement releasing chlorhexidine (Figs 3d and 4). The use of ALBC is a highly debated topic as often it is reported that the use of such material does not reduce the risk of PJI [34,35]. This is not totally surprising as antibiotic release only provides antimicrobial activity for about a week while the majority of potential PJI beyond the window of efficacy of ALBC; furthermore PJI isolates can also exhibit resistance to the antibiotics added to PMMA bone cement such as gentamicin or tobramycin; however, sustained release of non-antibiotic antimicrobial compounds could mitigate PJI risk for longer and thus justify the addition. The PJI isolates used here were all gentamicin resistant (MIC > 250 mg/l) and thus represent some of the most threatening strains; the ability of bone cement containing chlorhexidine to inhibit their growth and the extension of such property when the drug is released from silica nanocarriers mixed to the bone cement sample, highlight the potential benefits of the drug delivery presented in stopping potential PJIs.

The rheological behaviour of acrylic bone cements is very important for their mixing/handling and viscoelastic properties during the curing phase have significant influence on the cement porosity, degree of bone penetration and strength of the prosthesis/cement interface [36]. Curing time also determines the time available for the surgeon to position the dough in the required spaces to achieve fixation of the joint replacement device to the bone; therefore the introduction of nanoparticles into the cement formulation must not affect the rheological properties of the material. Dough time is defined as the time after mixing of the two components of the bone cement (liquid phase and solid phase) at which a freshly exposed cement surface fails to adhere to a powder-free latex glove. The setting time is defined as the time when the temperature of the cement reaches halfway between ambient and the peak exothermic temperatures (“ISO 5833:2002,” 2002). However, using viscoelastic parameters such as G’ (storage modulus), that corresponds to the elastic behaviour of the material, and G” (loss modulus), that corresponds to the viscous behaviour [37], provides a better and more objective description of bone cement handling and setting characteristics [38]. The profiles detected for G’ and G” observed (Fig 5a–5c) were comparable to those presented by others for PMMA bone cements [19,38]. The initial increase in viscosity is due to the swelling and dissolution of PMMA in liquid monomer, while the final rapid increase in viscosity is due to polymer formation [38]. It could have been expected nanoparticles to improve bone cement properties through the formation of a nanocomposite material; however this was not observed and likely due to the already high concentration of inert material (BaSO_4_) present in PMMA bone cements.

When in contact with body fluids, bone cement absorbs liquid; this uptake not only affects the mechanical properties of the bone cement, but it was also found to affect the surface properties and structure of the cement leading to a decrease in its molecular weight over long periods of time [39]. Thus, an initial determination of the water uptake behaviour is necessary to estimate the change in the physicochemical properties of the bone cement. Aging of bone cement in physiological conditions causes a decrease in the mechanical properties as result of the plasticising effect of water uptake that decreases attraction between polymer chains and increases flexibility [40]. Because Cemex material properties (Fig 6) meet regulations, it was not necessary to develop a material with superior performance but non-inferiority of bone cement containing the silica nanocarriers compared to Cemex was considered sufficient. The addition of chlorhexidine in the powder form is detrimental to the mechanical properties of the cement, while the nanoparticles loaded chlorhexidine preserves the cement mechanical properties. The morphology of fractured surfaces of ALBCs revealed cluster of antibiotic powder agglomerations, which may act as crack propagation points that weakens the cement mantle and decrease the mechanical properties [41]. Also, chlorhexidine is known to interfere with the free radical polymerization reaction of PMMA due to chlorhexidine free radical quenching effect [42]; deposition of chlorhexidine in multilayers coatings on nanoparticle seems to provide better mixing and less agglomeration inside the cement mantle, which can preserve the mechanical properties of the cement.

The potential adverse local and systemic consequences of chlorhexidine use are barely known, although, chlorhexidine has been shown cytotoxic to human osteoblasts, fibroblasts and lymphocytes in dose and time dependent manner [43–46]. Extensive chondrolysis was reported after accidental irrigation with 1% chlorhexidine solution during knee arthroscopy [47]; while chlorhexidine at a concentration of 0.2% was cytotoxic to Saos-2 cell and human gingival fibroblasts [48].

We assessed the impact of the new developed material on the viability, mitochondrial activity and metabolic markers of osteoblast cells encountered in arthroplasty sites.

Osteoblasts differentiation and commitment are controlled by complex genetic pathways; therefore, in order to elucidate the effect of chlorohexidine releasing multilayers we analysed different known markers expressed during the process of osteoblasts differentiation [49]. Ca deposition and ALP release are indicators of mineralization process [50]. Osteocalcin and osteopontin are the major non-collagen proteins involved in the processes bone matrix organization and deposition [51], while COLI expression is also involved in bone formation [52].

In this work, the concentrations of chlorhexidine released after the first day was ~300 μg/ml (0.3% w/v %) that is lower compared to the solution tested in above mentioned studies. The non-significant impact on mitochondrial activity (Fig 7) and metabolic markers (Figs 8 and 9) of osteoblasts observed here could be due to different activities of chlorhexidine when in PBS solution vs basal medium, as the later may exhibit a protective effect or support cell recovery. Moreover, the cells were grown in medium containing foetal bovine serum that has also a protective activity against chlorhexidine induced cytotoxicity [14]. In summary our in vitro findings showed that the developed bone cement formulation is safe to use.

## Conclusions

The application of multilayers nano-delivery systems may play a vital role in improving the release of non-antibiotic therapeutic agents such chlorhexidine from the bone cement, which is needed to reduce infection rates after TJRs. Also, it could provide a solution to the occurrence of PJI for longer periods of time and a solution to antimicrobial resistance without compromising other properties needed for performance.

## Supporting information

S1 Fig QQ-plot of size distribution of silica nanoparticles. (DOCX)

S1 Data NP characterisation dataset. (XLSX)

S2 Data Bone cement dataset. (XLSX)
