# Supplementary material for: PMMA bone cement containing long releasing silica-based chlorhexidine nanocarriers
Source: PLoS One. 2021 Sep 29;16(9):e0257947. doi: 10.1371/journal.pone.0257947 (PMC8480893; doi:10.1371/journal.pone.0257947)
Supplement: S1 Fig — (DOCX) [file pone.0257947.s001.docx]

*
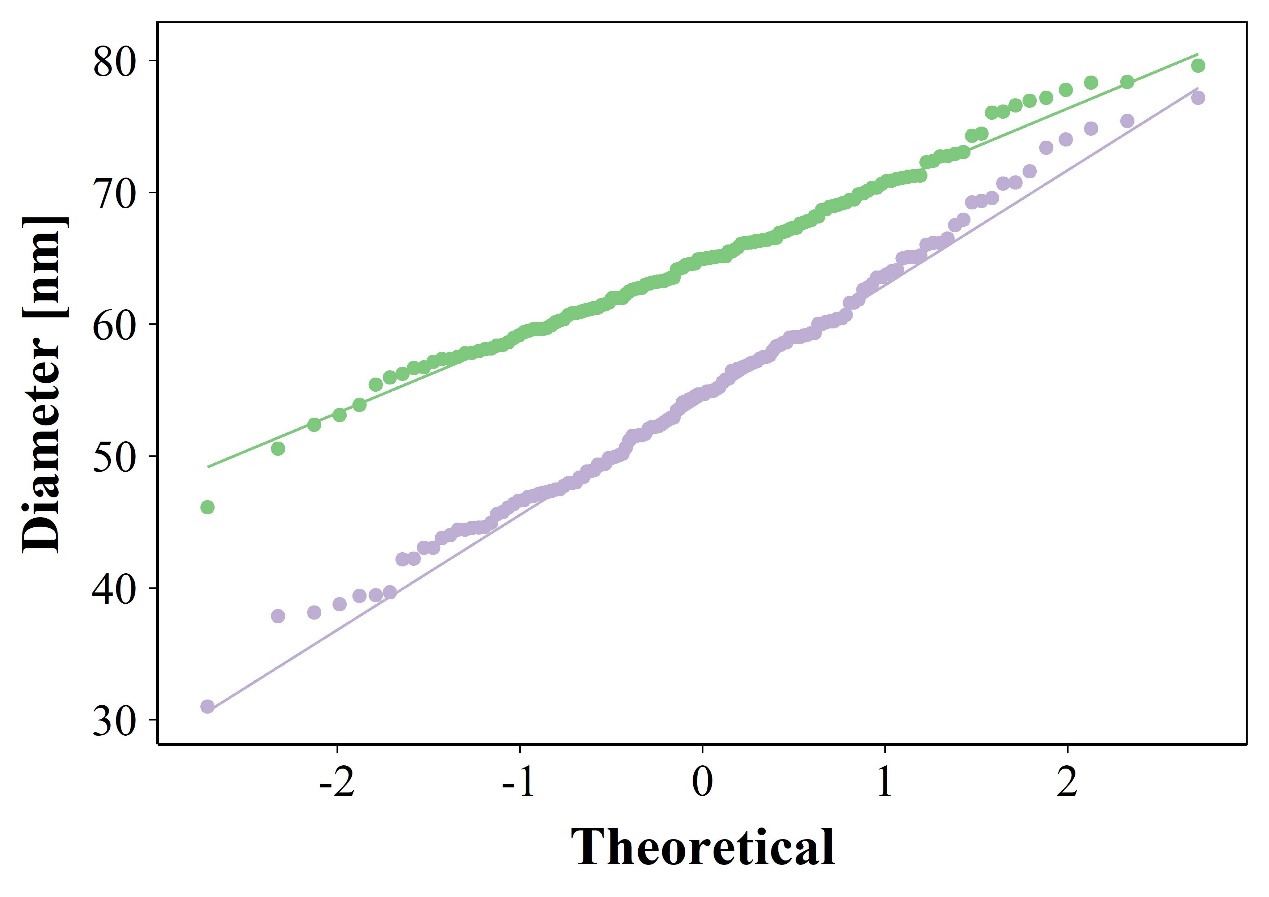
*

**S1 Figure. QQ-plot of size distribution of silica nanoparticles.** Amino functionalised(violet) and after the deposition of 10 QL (green).
